# Hypothalamus Transcriptome Reveals Key lncRNAs and mRNAs Associated with Fecundity in Goats

**DOI:** 10.3390/ani15050754

**Published:** 2025-03-06

**Authors:** Yingshi Wei, Caiye Zhu, Xiaoyun He, Mingxing Chu

**Affiliations:** 1 State Key Laboratory of Animal Biotech Breeding, Institute of Animal Science, Chinese Academy of Agricultural Sciences, Beijing 100193, China; weiyingshi_wys@163.com; 2 College of Animal Science and Technology, Gansu Agricultural University, Lanzhou 730070, China; zhucy@gsau.edu.cn

**Keywords:** goat, hypothalamus, follicular phase, luteal phase, high fecundity, lncRNA, mRNA

## Abstract

The hypothalamus (hyp) serves as the regulatory hub of the neuroendocrine system, synthesizing and secreting reproductive hormones that modulate estrus, follicular maturation, and embryonic development in goats. This study employed RNA-seq analysis to examine gene expression in the hypothalamic tissue of Yunshang black goats during the luteal phase in goats with high fecundity (LP_HY), during the luteal phase in goats with low fecundity (LP_LY), during the follicular phase in goats with high fecundity (FP_HY), and during the follicular phase in goats with low fecundity (FP_LY). Differential long non-coding RNAs (DE lncRNAs) and differential mRNAs (DE mRNAs) were subjected to Gene Ontology (GO) and Kyoto Encyclopedia of Genes and Genomes (KEGG) enrichment analyses and the construction of co-expression networks associated with reproduction. As a result, DE lncRNAs (390, 375, 405, and 394) and DE mRNAs (1836, 2047, 2003, and 1963) were identified in the four comparisons, namely FP_LY vs. FP_HY, LP_HY vs. FP_HY, LP_LY vs. FP_LY, and LP_LY vs. LP_HY, respectively. Functional annotations indicated significant enrichment of numerous DE lncRNAs and DE mRNAs in reproduction-related pathways such as the gonadotropin-releasing hormone pathway, the prolactin signaling pathway, the estrogen signaling pathway, the Wnt signaling pathway, oocyte meiosis, and progesterone-mediated oocyte maturation. The co-expression network of lncRNAs and target genes identified the interrelationships between reproduction-related genes such as *IGF1*, *PORCN*, *PLCB2*, *MAPK8*, *PRLR*, and *CPEB2* with our newly discovered lncRNAs. This study expands the understanding of lncRNAs and mRNAs in goat hypothalamic tissue and provides new insights into molecular mechanisms related to goat reproduction.

## Simple Summary

This study aimed to understand how specific genes in the goat’s hypothalamus, including long non-coding RNAs (lncRNAs) and messenger RNAs (mRNAs), affect goat fertility. We looked at gene expression patterns in goats with high and low fertility during different stages of their reproductive cycles. Our results showed that many lncRNAs and mRNAs are involved in reproductive hormone pathways and thus could influence the number of offspring goats can produce. This research could help improve goat breeding programs by identifying key genetic factors that control fertility.

## 1. Introduction

With their extensive distribution, goats are a significant economic resource, offering valuable products such as meat, milk, and fiber to human societies [1]. The reproductive capacity of goats is a critical economic determinant [2]. Reproductive capacity in goats is influenced by age, nutrition, environmental conditions, husbandry practices, and genetic factors such as gene expression and mutations [3,4]. Transcriptomics offers profound insights into gene expression profiles and regulatory pathways [5], thereby providing crucial leads for dissecting the molecular underpinnings of reproductive efficiency.

The hypothalamus, as the central hub of the neuroendocrine system, plays a vital role in regulating estrous cycles and follicular development in goats [6]. The hypothalamus synthesizes gonadotropin-releasing hormone (GnRH), which facilitates the release of luteinizing hormone (LH) and follicle-stimulating hormone (FSH) from the anterior pituitary [7]. These hormones are crucial for the development of oocytes, the growth and maturation of follicles, the process of ovulation, and the formation and subsequent regression of the corpus luteum [8,9]. Thus, the hypothalamus is paramount in orchestrating the reproductive capabilities of goats.

Long non-coding RNAs (lncRNAs), a category of transcripts longer than 200 nucleotide pairs, do not contribute to the coding of proteins [10]. There is evidence that lncRNAs control the expression of protein-coding genes [11,12]. Numerous studies have shown that lncRNAs play an important role in the modification and regulation of gonadal development and reproduction in animals. For example, the overexpression of lncRNA-Gm2044 within spermatogonial cells has been shown to suppress spermatogenesis in select seminiferous tubules [13]. The lncRNA TUNAR is implicated in the human endometrial milieu, potentially participating in the processes of embryo implantation, proliferation of endometrial stromal cells, and decidualization [14]. Additionally, Lnc107153 is involved in the regulatory control of CHGA expression, influencing the seasonal reproductive patterns observed in sheep [15]. The lncRNA designated as XR_001039544.4 is hypothesized to modulate the secretion of luteinizing hormone (LH) in the pituitary during the luteal phase, potentially through its impact on cellular proliferation [16].

LncRNAs have been found to participate in a spectrum of reproductive processes such as oogenesis [17], follicular growth [18], oocyte maturation [19], ovulation [20], embryonic development [21], pregnancy [22], and the determination of sex [23]. In a recent study, lncRNAs were identified as integral to the modulation of reproductive processes in the hypothalamus of goats [24]. Nevertheless, the study of lncRNAs mainly focuses on differential expression in specific tissues in animals with different reproductive capacity levels [25,26]. Some studies have also investigated the differential expression of lncRNAs during the follicular and luteal phases in other species, such as sheep [27] and pigs [28]. Current research on the hypothalamus has focused on groups differing in reproductive capacities [24], overlooking the importance of the follicular and luteal phases in fertility. Therefore, in this study, we analyzed the expression of reproduction-related lncRNAs and mRNAs in the hypothalamuses of goats with different reproductive abilities at different physiological stages using RNA-Seq, aiming to reveal the link between reproductive mRNAs and novel DE lncRNAs by constructing co-expression networks.

## 2. Materials and Methods

### 2.1. Ethics Statement

The animal study, under a permit (No. IAS2019–63), was reviewed and approved by the Institutional Animal Care and Use Ethics Committee of the Institute of Animal Sciences and Chinese Academy of Agricultural Sciences (IAS-CAAS) (Beijing, China) for all of the experimental procedures mentioned.

### 2.2. Animals and Sample Collection

All Yunshang black goats (3 years old) were sourced from the Yixing Heng Animal Husbandry Technology Co., Ltd. Unity Township Base, Yunnan Province, China (high-fecundity group, *n* = 10, mean lamb number 3.4 ± 0.42, HY group; low-fecundity group, *n* = 10, mean lamb number 1.8 ± 0.27, LY group). They were housed under uniform environmental conditions and, before the study, received synchronized estrous treatment via a progesterone vaginal suppository (CIDR). Following a 16-day interval post-CIDR insertion, the devices were removed and the goats were humanely euthanized with an overdose of pentobarbital sodium (1.5 mg/kg) at distinct temporal points relative to their reproductive phases, specifically, within 45–48 h post-CIDR removal for the follicular phase (FP) and within 168 h for the luteal phase (LP). The goats were categorized into quartets based on their lambing records and estrous cycle patterns: high-fecundity goats in the luteal phase (*n* = 5, LP_HY group), low-fecundity goats in the luteal phase (*n* = 5, LP_LY group), high-fecundity goats in the follicular phase (*n* = 5, FP_HY group), and low-fecundity goats in the follicular phase (*n* = 5, FP_LY group). The hypothalamic tissues were promptly harvested post-euthanasia, preserved in liquid nitrogen, and subsequently transported to the laboratory for storage at −80 °C.

### 2.3. Total RNA Extraction and Library Construction

Total RNA was isolated from the cryopreserved hypothalamic tissues of 20 goats using TRIzol reagent (Invitrogen, Carlsbad, CA, USA). RNA concentration and purity were quantified and assessed with a NanoDrop 2000 spectrophotometer (Thermo Scientific, Wilmington, DE, USA). The integrity of the extracted RNA was confirmed through 1.5% agarose gel electrophoresis and further validated using an Agilent 2100 Bioanalyzer (Agilent Technologies, Santa Clara, CA, USA) for a comprehensive evaluation of RNA integrity. The rRNA was depleted using the Epicentre Ribo-zero™ rRNA Removal Kit (Epicentre, Madison, WI, USA). The libraries were constructed using the NEB Next Ultra™ Directional RNA Library Prep Kit for Illumina (NEB, Beijing, China). Subsequently, the libraries were sequenced on the Illumina NovaSeq platform (150 bp paired-end reads, Illumina, Hayward, CA, USA) at Wuhan Frasergen Bioinformatics Co., Ltd. (Wuhan, China).

### 2.4. Sequencing Data Processing and Transcriptome Assembly

The raw reads in fastq format were filtered using SOAPnuke (v2.1.0) to remove reads containing adapter sequences, reads with poly-N bases accounting for more than 1% of all bases, and low-quality paired-end reads. Subsequently, the Phred quality score was used to assess the sequencing error rates, and the Q20, Q30, and GC content of the filtered data were obtained. We utilized HISAT2 (v.2.1.0) to map the clean reads to the reference genome (GCF_001704415.2). For transcript assembly, we employed StringTie (v.1.3.5) software, assembling only uniquely mapped reads. The fragments per kilobase of the exon model per million mapped fragments (FPKM) for each gene were determined.

### 2.5. lncRNA Identification and Differential Expression Analysis

Candidate lncRNAs (lincRNAs, intronic lncRNAs, and anti-sense lncRNAs) were screened based on the locations of the encoded reads. Only transcripts with a length of at least 200 base pairs and multiple exons were selected for further analysis. Transcripts corresponding to known mRNAs and other non-coding RNA species, such as ribosomal RNAs (rRNAs) and transfer RNAs (tRNAs), were filtered out using Cuffcompare. The final set of novel lncRNAs was determined by the intersection of predictions from the CNCI (v.2.0), CPC2 (v.1.0.1), and PLEK (v.1.2) software tools.

This study included five biological replicates, with transcript expression levels quantified by FPKM values. Before performing differential expression analysis, we conducted Shapiro−Wilk normality tests and Levene’s tests for homogeneity of variance on the FPKM values of both mRNAs and lncRNAs. The results indicated that the mRNA data generally satisfied the assumptions of normality and homogeneity of variance, while the lncRNA data in the LP_HY and FP_LY groups did not meet the normality assumption (Supplementary Table S8). Therefore, we applied the non-parametric method DESeq2 for lncRNA analysis to ensure robust results. Transcripts with an absolute log2 fold change (|Log2FC|) greater than 1 and a *p*-value less than 0.05 were identified as significantly differentially expressed.

### 2.6. GO and KEGG Enrichment Analysis

The relationship between lncRNAs and potential protein-coding genes was predicted based on the distance and co-expression of lncRNAs. The target genes 100 kb upstream and downstream of each lncRNA were identified as potential cis-elements. The Pearson correlation coefficient method was used to analyze the correlations between lncRNAs and mRNAs among samples. The mRNAs with absolute values of correlation greater than 0.95 were used as trans-target genes for functional enrichment analysis to predict the main functions of the lncRNAs. The online software Metascape (https://metascape.org/gp/index.html, accessed on 5 July 2024) was used for GO and KEGG pathway enrichment analysis.

### 2.7. Target-Gene Prediction and Network Construction of lncRNA

The lncRNA−mRNA network was constructed based on the targeting relationship, and the lncRNA and its target gene network were visualized using Cytoscape software (v.3.10.1, Cytoscape Consortium, San Diego, CA, USA).

## 3. Results

### 3.1. Overview of Hypothalamus Transcriptome Sequencing Data

From the LP_HY, LP_LY, FP_HY, and FP_LY groups, five cDNA libraries were independently constructed for next-generation sequencing (group details provided in Supplementary Table S1). Each sample produced over 14.8 G of clean reads, with mean Q20 and Q30 values of 98.4%, 97.8%, 95.2%, and 93.6%, respectively. The average GC contents were 46.6% and 47.1%. The mean percentages of total and uniquely mapped reads across all samples were 96.51% and 90.26%, respectively. Further details are available in Supplementary Table S2.

### 3.2. Identification of lncRNA and mRNA

**Figure 1:**
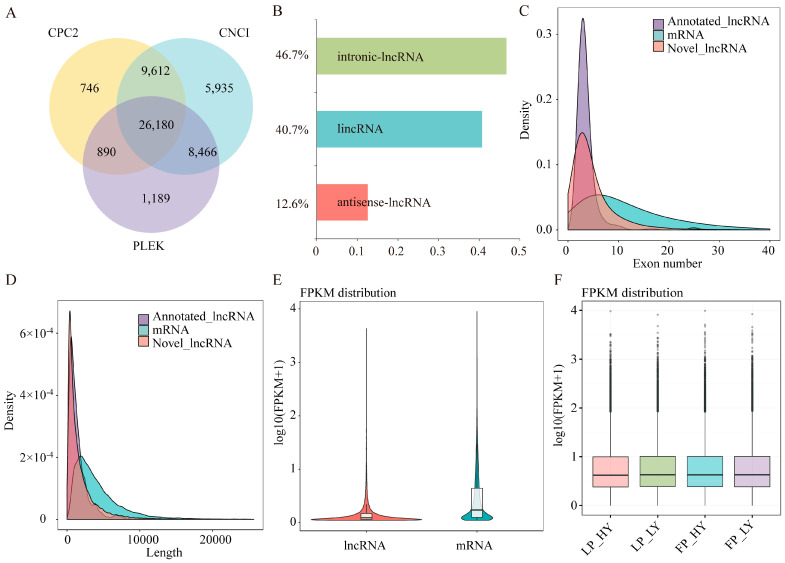
The expression profiles of lncRNAs and mRNAs in the hypothalamuses of goats. (**A**) The Venn diagram represents the collective and distinct numbers of lncRNAs identified through coding predictions by CNCI, CPC2, and PLEK. (**B**) Classification of novel lncRNAs: lincRNA, antisense lncRNA, and intronic lncRNA. (**C**) Exon-number statistics for mRNAs, novel lncRNAs, and annotated lncRNAs. (**D**) Length statistics for mRNAs, novel lncRNAs, and annotated lncRNAs. (**E**) The expression levels of lncRNA transcripts and mRNA transcripts. (**F**) The expression-level distributions of lncRNAs in different experimental groups.

By utilizing the CNCI, CPC2, and PLEK tools, we identified 26,180 novel lncRNA loci and 1769 annotated lncRNAs, as outlined in Supplementary Table S3 (Figure 1A). Intronic lncRNAs were the most prevalent, accounting for 46.7% of the total, followed by lincRNAs at 40.7% and anti-sense lncRNAs at the lowest proportion, 12.6% (Figure 1B). The length of the majority of lncRNAs ranged between 200–3000 base pairs (mean 1839 bp), with exon counts typically ranging from 2–8 (mean 4.8). In comparison, mRNAs were mainly distributed between 1000–6000 base pairs (mean 4458 bp), with exon counts typically between 2–15 (mean 12) (Figure 1C,D, Supplementary Table S4). Expression analysis using Log10(FPKM+1) indicated that mRNA transcripts exhibited higher expression levels in the hypothalamus than did lncRNA transcripts (Figure 1E). The expression levels of transcripts under various experimental conditions were largely consistent across samples (Figure 1F, Supplementary Table S5).

### 3.3. Analysis of DE lncRNAs and DE mRNAs

**Figure 2:**
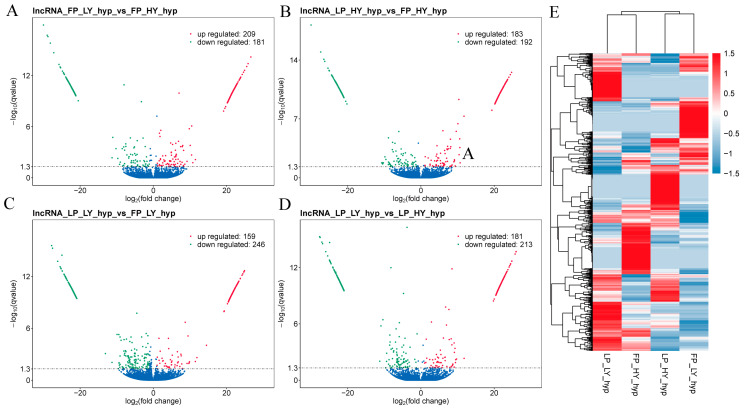
Expression analysis of DE lncRNAs. (**A**) Volcano diagram of DE lncRNAs in the comparison FP_LY vs. FP_HY. (**B**) Volcano diagram of DE lncRNAs in the comparison LP_HY vs. FP_HY. (**C**) Volcano diagram of DE lncRNAs in the comparison LP_LY vs. FP_LY. (**D**) In the volcanogram of DE lncRNAs in the comparison LP_LY vs. LP_HY, the horizontal line represents q-value < 0.05; red represents up-regulation and green represents down-regulation. (**E**) Hierarchical clustering analysis of DE lncRNAs in the FP_HY, FP_LY, LP_HY, and LP_LY groups shows that the intensity increased from blue to red, with these colors indicating down-regulation and up-regulation, respectively.

**Figure 3:**
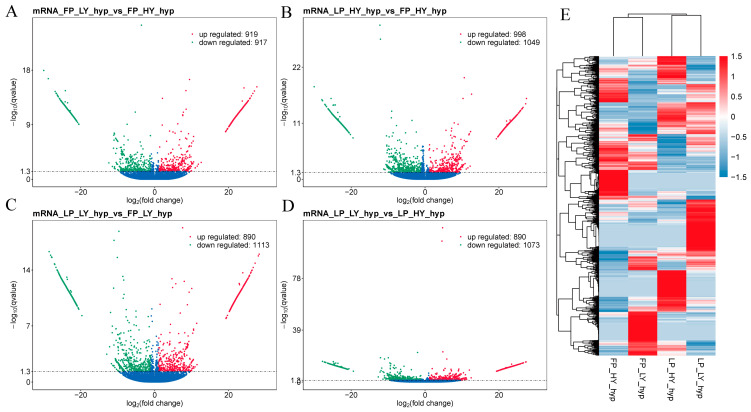
Expression analysis of DE mRNAs. (**A**) Volcano diagram of DE mRNAs from the comparison FP_LY vs. FP_HY. (**B**) DE mRNA volcano plot from the comparison LP_HY vs. FP_HY. (**C**) Volcano diagram of DE mRNAs from the comparison LP_LY vs. FP_LY. (**D**) DE mRNA volcano plot from the comparison LP_LY vs. LP_HY, the horizontal line represents q-value < 0.05; with red representing up-regulation and green representing down-regulation. (**E**) Hierarchical cluster analysis of DE mRNAs in the FP_HY, FP_LY, LP_HY, and LP_LY groups shows that the intensity increased from blue to red, with these colors indicating down-regulation and up-regulation, respectively.

In the comparison FP_LY vs. FP_HY, 390 DE lncRNAs (209 up-regulated and 181 down-regulated, Figure 2A) and 1836 DE mRNAs (919 up-regulated and 917 down-regulated, Figure 3A) were identified. A total of 375 DE lncRNAs (83 up-regulated and 192 down-regulated, Figure 2B) and 2047 DE mRNAs (998 up-regulated and 1049 down-regulated, Figure 3B) were identified in the comparison LP_HY vs. FP_HY. In the comparison LP_LY vs. FP_LY, a total of 405 DE lncRNAs (159 up-regulated and 246 down-regulated, Figure 2C) and 2003 DE mRNAs (890 up-regulated and 1113 down-regulated, Figure 3C) were identified. In the comparison LP_LY vs. LP_HY, a total of 394 DE lncRNAs (181 up-regulated and 213 down-regulated, Figure 2D) and 1963 DE mRNAs (890 up-regulated and 1073 down-regulated, Figure 3D) were identified. Detailed information on the expression of DE lncRNAs between groups is given in Supplementary Table S6, and detailed information on the expression of DE mRNAs is shown in Supplementary Table S7. 

To further evaluate the biological significance of the observed differences, we calculated effect sizes (|Cohen’s d|) for DE mRNA and DE lncRNA comparisons between groups. The effect sizes for DE mRNA comparisons ranged from 0.798 to 1.324, with the largest effect observed in the LP_LY vs. FP_LY comparison. For DE lncRNA comparisons, effect sizes ranged from 0.438 to 0.891, indicating moderate-to-large effects in several groups (Supplementary Table S8). These results suggest that the observed differences are not only statistically significant but also biologically meaningful.

A hierarchical clustering analysis of DE lncRNAs and DE mRNAs in the FP_HY, FP_LY, LP_HY, and LP_LY groups was conducted, and a heatmap was generated. The visualization method was employed to further investigate the rationality of the grouping (refer to Figure 2E and Figure 3E, Supplementary Table S9). When DE mRNAs were analyzed, many genes that have been validated to be associated with reproduction were identified, such as *IGF1*, *PORCN*, *PRLR*, *MAPK8*, *CPEB2*, *CPEB3*, *ATF6B*, *PLCB2*, *PPP1CB*, and *CREB3L4*.

### 3.4. GO and KEGG Enrichment Analysis of DE mRNA

**Figure 4:**
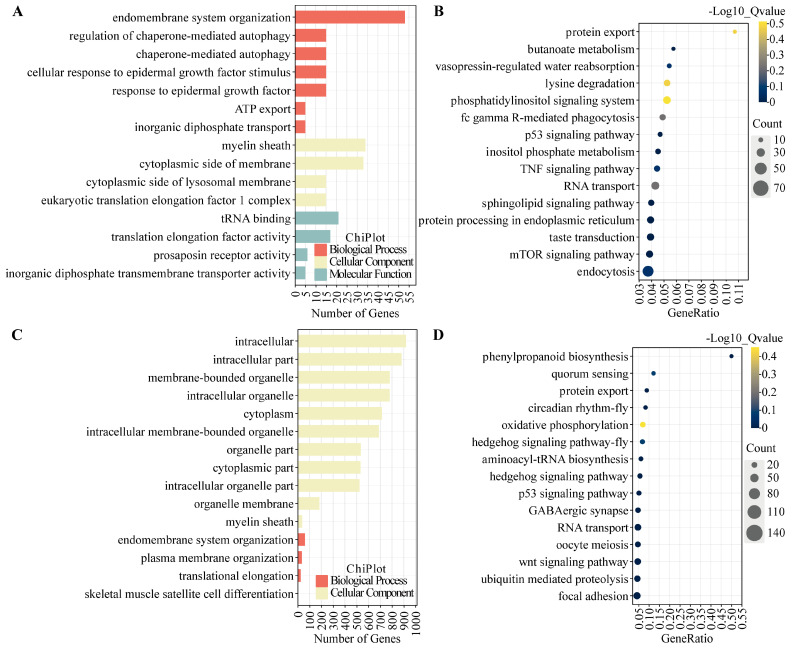
GO and KEGG enrichment analysis of DE mRNAs in the hypothalamus. (**A**) GO functional analysis of DE mRNAs in the comparison FP_LY vs. FP_HY. (**B**) KEGG enrichment pathway of DE mRNAs in the comparison FP_LY vs. FP_HY. (**C**) GO function analysis of DE mRNAs in the comparison LP_HY vs. FP_HY. (**D**) KEGG enrichment pathway of DE mRNAs in the comparison LP_HY vs. FP_HY.

**Figure 5:**
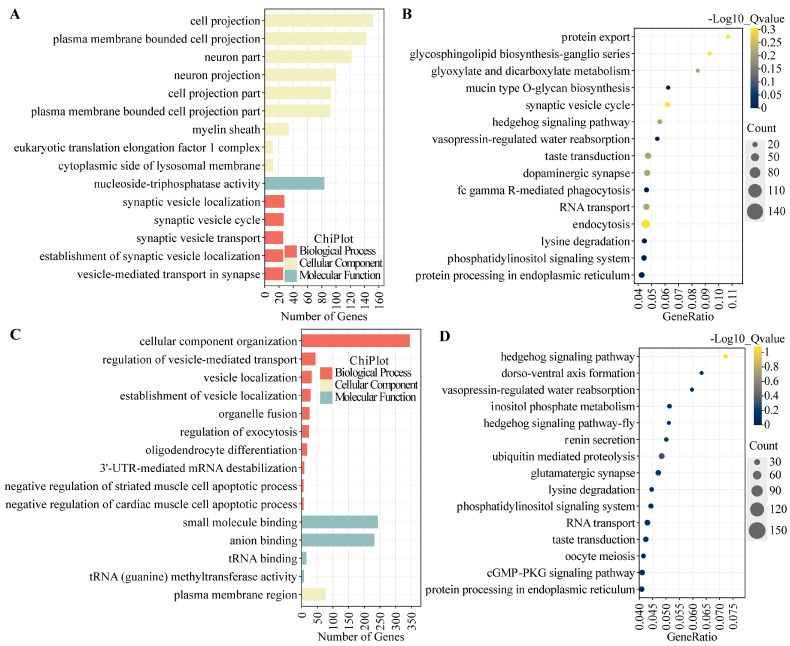
GO and KEGG enrichment analysis of DE mRNAs in the hypothalamus. (**A**) GO function analysis of DE mRNAs in the comparison LP_LY vs. FP_LY. (**B**) KEGG enrichment pathway of DE mRNAs in the comparison LP_LY vs. FP_LY. (**C**) GO function analysis of DE mRNAs in the comparison LP_LY vs. LP_HY. (**D**) KEGG enrichment pathway of DE mRNAs in the comparison LP_LY vs. LP_HY.

Target genes of differentially expressed (DE) mRNAs underwent Gene Ontology (GO) and Kyoto Encyclopedia of Genes and Genomes (KEGG) enrichment analyses, the results of which are presented in Figure 4 and Figure 5 and detailed in Supplementary Table S10. Significant GO terms were identified within the Biological Process (BP), including response to epidermal growth factor, synaptic vesicle localization, and regulation of vesicle-mediated transport. Molecular Function (MF) categories encompass small-molecule binding, tRNA binding, and prosaposin receptor activity. Cellular Component (CC) terms encompass neuron projection, cell projection, and plasma membrane-bounded cell projection. These enrichments are largely associated with neural signal transduction and are hypothesized to contribute to the modulation of reproductive behaviors and hormonal secretions.

KEGG pathway enrichment analysis identified the 15 most significantly enriched pathways. Of particular interest, oocyte meiosis was prominent among the enriched DE mRNAs across all experimental groups, as referenced in Figure 4 and Figure 5 (not displayed in Figure 4B and Figure 5B; comprehensive details are presented in Supplementary Table S11). In the LP_HY vs. FP_HY comparison, reproduction-related pathways included the Wnt signaling pathway, circadian rhythm in flies, the p53 signaling pathway, the Hedgehog signaling pathway in flies, quorum sensing, and ubiquitin-mediated proteolysis (Figure 4D). When LP_LY vs. LP_HY were contrasted, the reproduction-associated pathways identified comprised dorso-ventral axis formation, the phosphatidylinositol signaling system, ubiquitin-mediated proteolysis, and the Hedgehog signaling pathway (Figure 5D). In the FP_LY vs. FP_HY analysis, enrichment was observed for the p53 signaling pathway and the phosphatidylinositol signaling system (Figure 4B). Between LP_LY vs. FP_LY, the Hedgehog signaling pathway and the phosphatidylinositol signaling system were found to be enriched (Figure 5B).

### 3.5. Construction of lncRNA and mRNA Co-Expression Network

The co-expression network of novel DE lncRNAs and known target DE mRNAs between groups was constructed. It was observed that there was a positive correlation between lncRNAs and mRNAs in each group. The networks were based on the Pearson correlation coefficient (PCC). The LP_HY vs. FP_HY group was subjected to a stringent cutoff (absolute PCC value ≥ 0.4, *p*-value < 0.05), while the remaining groups were subjected to a more lenient threshold (absolute PCC value ≥ 0.5, *p*-value < 0.05).

**Figure 6:**
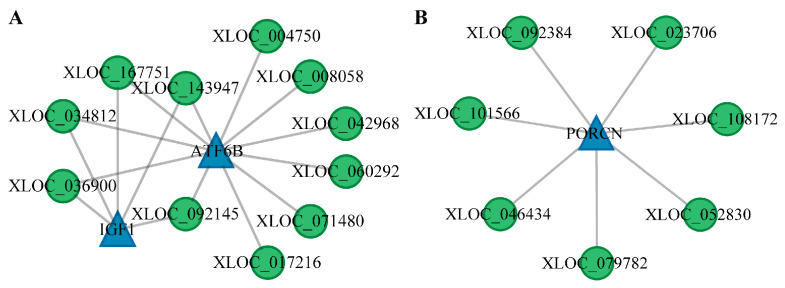
The co-expression networks of lncRNAs and mRNAs. (**A**) The co-expression network of DE lncRNA and target DE mRNAs in FP_LY vs. FP_HY. (**B**) The co-expression network of DE lncRNAs and target DE mRNAs of LP_HY vs. FP_HY. The blue triangle represents lncRNAs, and the green circle represents mRNAs; the same below.

**Figure 7:**
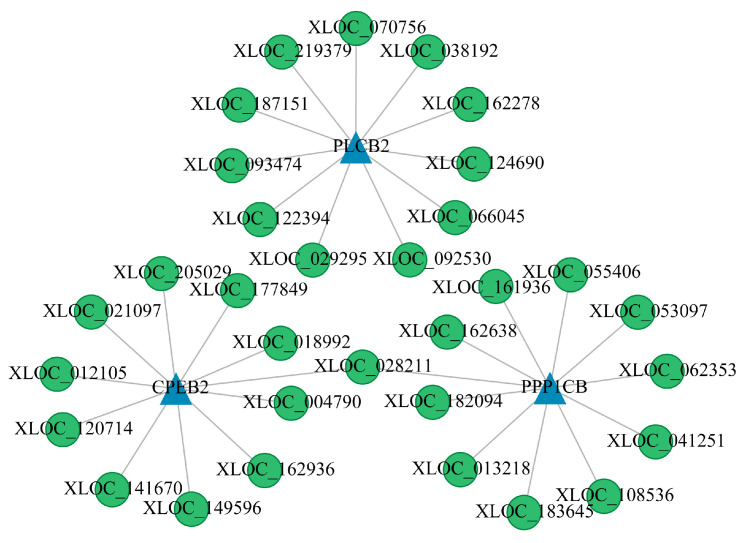
The co-expression network of DE lncRNA and target DE mRNAs in the comparison LP_LY vs. LP_HY was constructed.

**Figure 8:**
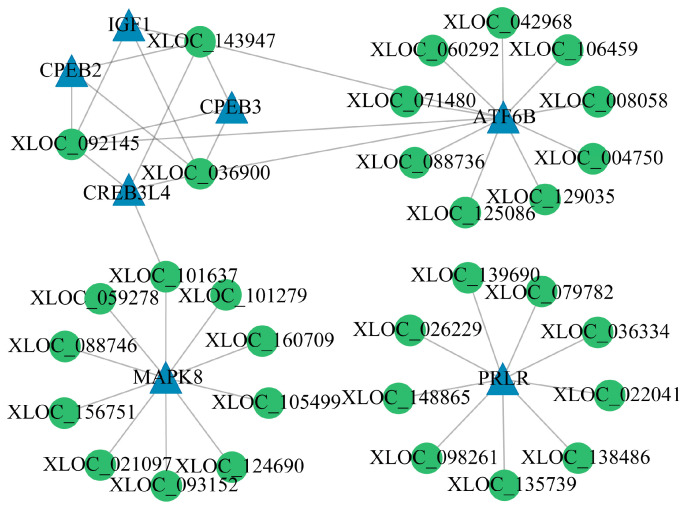
The co-expression network of DE lncRNAs and target DE mRNAs in the comparison LP_LY vs. FP_LY.

The co-expression network for the FP_LY vs. FP_HY comparison consists of 13 nodes and 16 edges, including associations of XLOC-034812, XLOC-036900, XLOC-092145, XLOC-143947, and XLOC-167751 with *IGF1* and *ATF6B* (Figure 6A). In the LP_HY vs. FP_HY comparison, the network comprises eight nodes and seven edges, highlighting the relationship of *PORCN* with seven novel lncRNAs (Figure 6B). The LP_LY vs. LP_HY comparison shows a network of 38 nodes and 44 edges, with XLOC-028211 correlated to *CPEB2* and *PPP1CB* (Figure 7). The LP_LY vs. FP_LY comparison reveals a network of 35 nodes and 33 edges, with XLOC-036900, XLOC-092145; XLOC-143947 linked to *IGF1*, *ATF6B*, *CREB3L4*, *CPEB2*, and *CPEB3*; and XLOC-101637 associated with *MAPK8* and *CREB3L4* (Figure 8). Detailed information on these lncRNA−mRNA networks is available in Supplementary Table S12.

## 4. Discussion

Reproduction in mammals is coordinated by the hypothalamic-pituitary-gonadal (HPG) axis. GnRH, originating from the hypothalamus, regulates the synthesis and secretion of the pituitary gonadotropins FSH and LH and plays a crucial role in the control of reproduction [29]. This study discovered a significant number of mRNAs in various pathways, including the GnRH signaling pathway, prolactin signaling pathway, estrogen signaling pathway, Wnt signaling pathway, oocyte meiosis pathway, and progesterone-mediated oocyte maturation pathway. These findings are consistent with the results of Hou et al. [24] regarding the hypothalamic tissue of Chuanzhong goats with different reproductive capacities. Dong et al. [30] identified that the prolactin signaling pathway in the hypothalamus of Leizhou goats could impact litter size. Similarly, research on the bovine hypothalamus has shown a notable enrichment of target genes of DE lncRNAs in pathways related to secretion of reproductive hormones, such as the GnRH signaling pathway [31]. For example, in the LP_LY vs. FP_LY comparison, *IGF1*, a critical factor for mammalian follicular development [32], was enriched in the ovarian steroidogenesis pathway. *CAMK2G*, *ITPR2*, *MAPK8*, and *CACNA1C* are closely associated with neural transmission and signal-transduction processes, showing significant enrichment within the GnRH signaling pathway. Studies have shown that the GnRH signaling pathway regulates the reproductive process by mediating different frequencies of GnRH pulses to cause changes in the secretion patterns of FSH and LH [33,34]. *PIK3* is involved in trophoblast differentiation, extravillous trophoblast invasion, and migration [35], *ESR1* and *PRLR* are also essential factors that regulate reproduction. *ESR1*, *PIK3CG*, and *PIK3CD* are enriched in both the prolactin signaling pathway and the estrogen signaling pathway, while *PRLR* exhibits specific enrichment in the prolactin signaling pathway.

The co-expression analysis of lncRNAs and mRNAs suggests the existence of complex interactions, ranging from one-to-many to many-to-one relationships. Within the FP_LY vs. FP_HY comparison, a positive correlation was observed between the mRNAs *IGF1* and *ATF6B* and the lncRNAs XLOC-034812, XLOC-036900, XLOC-092145, XLOC-143947, and XLOC-167751. Corroborating research has identified *IGF1* as a well-established regulatory factor in mammalian follicular development that synergizes with FSH to potentiate its effects [32,36]. In pre-ovulatory follicles of avian species, *IGF1* upregulates the expression of genes implicated in steroidogenic processes and the biosynthesis of progesterone and inhibin A [37]. Mani et al. found that IGF1 induces up-regulation of steroidogenic and apoptotic regulatory genes via activation of phosphatidylinositol-dependent kinase/AKT in bovine granulosa cells [32]. Research on induced ovulation in camels has identified a correlation between circulating levels of *IGF1* and circulating levels of estrogen (E2) [38]. Consequently, it is posited that a spectrum of lncRNAs, with *IGF1* as their target gene, partake in the modulation of an array of reproductive hormones. The activity of *IGF1* is conveyed through its cell surface receptor, the insulin-like growth factor 1 receptor (IGF1R), which is localized on theca and granulosa cells of pre-ovulatory follicles in hens, with its expression levels rising in tandem with follicle enlargement [39]. Mice with inactivation of IGF1R in granulosa cells are rendered infertile due to the stagnation of follicular development in the pre-ovulatory phase [40]. In the transcriptomic and proteomic investigation of postpartum ovarian cyclicity cessation in yaks by Huo et al. [41], potential effectors regulating postpartum anestrus in yaks were identified, and the involvement of genes such as *ATF6B*, as well as that of calmodulins, in governing the status of the luteal phase in the postpartum regulation of estrus was confirmed. This implicates lncRNAs, including XLOC-034812, as possibly being related to the modulation of reproductive hormones, including progesterone, FSH, and E2.

In the comparison between LP_HY vs. FP_HY, *PORCN* was found to be the target gene of seven lncRNAs, including XLOC-092384, and was enriched in the Wnt signaling pathway. Harwood et al. [42] confirmed the expression of various transcripts and proteins required for WNT pathway activity in developing ovaries, oocytes and preimplantation embryos by RT-PCR and immunofluorescence assay, with results indicating that the Wnt signaling pathway plays an important role in follicular development. The PORCN protein is integral to the Wnt signaling cascade, enabling the secretion of Wnt proteins essential for ectodermal tissue development [43]. The Wnt signaling pathway, modulated by a repertoire of 19 Wnt ligands, serves as a crucial driver for stem cells within the majority of adult mammalian tissues [44]. These Wnt ligands have been demonstrated to be pivotal in the embryonic development of mammals, with *PORCN* influencing the function of all Wnt ligands [45]. The enzyme *PORCN* facilitates the palmitoylation of Wnt proteins on serine residues, thereby enhancing their secretion, a process critical for Wnt signal transduction and proper secretion mechanisms [46]. Research by Steffen et al. [47] has established that *PORCN* is essential for the Wnt3-mediated induction of primitive streak formation within embryonic tissues. This implies that a set of seven lncRNAs, with XLOC-092384 among them, may affect embryonic development by preserving the integrity of the Wnt signaling pathway and modulating the secretion of Wnt proteins.

Furthermore, within the LP_LY vs. LP_HY comparison, *CPEB*, *PLCB2*, and *PPP1CB* were each identified to regulate a subset of 11 lncRNAs, with the lncRNA XLOC-028211 being subject to concurrent regulation by *CPEB2* and *PPP1CB*. *CPEB* can regulate physiological processes such as germ cell development, cell division, and cell differentiation by regulating the translation process [48,49]. The *CPEB2* gene, a member of the *CPEB* family, facilitates meiotic maturation in porcine oocytes and promotes embryonic development [50]. Furthermore, *CPEB2* is crucial for the successful completion of mitotic cell division [51]. *PPP1CB* is imperative for the maturation of oocytes [52] and has been correlated with the germinal vesicle breakdown, as well as with chromatin condensation during metaphase I and II in murine oocytes [53]. These associations imply that the lncRNA XLOC-028211 could be implicated in the processes of follicular development and maturation. Investigations have confirmed that the expression of the *PLCB2* gene is upregulated in the choroid plexus of ovariectomized female rats, and that, conversely, the expression is downregulated by estrogen and progesterone [54]. These findings indicate that a cohort of 11 lncRNAs, XLOC-070756 among them, that interact with *PLCB2* may be correlated with the secretion of GnRH, OXT, and E2. Such interactions could be pivotal for the regulation of estrous-cycle stability and of fecundity.

Among the four groups examined, the comparison between the LP_LY and FP_LY groups yielded the most extensive repertoire of target genes associated with reproductive functions. XLOC-036900, XLOC-092145, and XLOC-143947 emerge as crucial nodes within this network, with their associated target genes including *IGF1*, *ATF6B*, *CREB3L4*, *CPEB2*, and *CPEB3*. The pivotal roles of *ATF6B*, *IGF1*, *CPEB2,* and *CPEB3* in regulating reproductive processes have been elucidated through comparative analyses of FP_LY vs. FP_HY and LP_LY vs. LP_HY. The network also highlights two crucial genes, *MAPK8* and *PRLR*, with *MAPK8* acting as a regulator of 10 lncRNAs, including XLOC-101637. The *MAPK8* gene has also been identified as a key gene associated with the fertility of multiparous sheep [55]. Research by Wang et al. [56] involved inhibiting or activating the c-Jun N-terminal kinase (JNK) signaling pathway by knocking out or overexpressing *MAPK8*, respectively, affecting the differentiation of embryonic stem cells. The *PRLR* regulates a subset of nine lncRNAs, with XLOC-139690 being one of them. The interaction between *PRLR* and *PRL* plays a crucial role in mediating biological effects and is involved in various reproductive pathways, significantly impacting reproductive capability [57]. The associations of *PRLR* with reproductive traits have been extensively documented across various species, including goats [58], sheep [59], pigs [60], and chickens [61]. Overall, the DE lncRNAs and respective target genes identified across the four group comparisons likely work together to modulate the hypothalamic regulation of reproductive functions.

In summary, the DE lncRNAs and their target genes, as identified in our study, may synergistically regulate the hypothalamus to affect the reproductive process of goats. However, the study also has shortcomings in that it is limited to specific goat breeds, which limits the generalisability of the findings. In addition, we relied mainly on transcriptional-level analyses and did not carry out in-depth functional validation of the identified molecules. In the future, we will carry out relevant functional validation of the identified DE lncRNAs to gain a more comprehensive understanding.

## 5. Conclusions

This study identified key lncRNAs and mRNAs associated with different physiological stages and fecundity levels in the goat hypothalamus, highlighting their potential role in reproductive regulation. These findings highlight the importance of these molecules in regulating the reproductive process and lay the foundation for further exploration. Future studies should focus on functional verification of identified molecules and their interactions to elucidate their exact roles in hypothalamic reproductive regulation. Such work will provide new insights into how to improve the reproductive efficiency of livestock.

## Data Availability

Due to privacy and ethical considerations, the sequencing data generated in this study have not been deposited into a publicly accessible database. These data contain sensitive information that could potentially compromise the confidentiality of the study participants. Consequently, the data are not publicly available. However, interested researchers may contact the corresponding author to discuss potential data sharing arrangements.

## Supplementary Materials

The following supporting information can be downloaded at: https://www.mdpi.com/article/10.3390/ani15050754/s1, Table S1: Sample grouping information; Table S2: The information of RNA-seq data; Table S3: CNCI, CPC2 and PLEK detection information; Table S4: mRNA, novel lncRNA and annotated lncRNA information; Table S5: The distribution of lncRNA expression levels in different samples; Table S6: Details of DE lncRNA expression between groups; Table S7: Details of mRNA expression between groups; Table S8: Shapiro-Wilk test, Levene’s test and Cohen’s d value information; Table S9: Hierarchical clustering information of DE lncRNA and DE mRNA in different experimental groups.; Table S10: GO enrichment analysis information; Table S11: KEGG pathway enrichment analysis information; Table S12: Co-expression network information of lncRNA and mRNA.
